# Sustained antiproliferative mechanisms by RB24, a targeted precursor of multiple inhibitors of epidermal growth factor receptor and a DNA alkylating agent in the A431 epidermal carcinoma of the vulva cell line

**DOI:** 10.1038/sj.bjc.6602098

**Published:** 2004-09-07

**Authors:** R Banerjee, Z Rachid, Q Qiu, J P McNamee, A M Tari, B J Jean-Claude

**Affiliations:** 1 Cancer Drug Research Laboratory, Department of Medicine, Division of Medical Oncology, McGill University Health Center/Royal Victoria Hospital, 687 Pine Avenue West, Rm. M 7.15, Montreal, Quebec, Canada H3A 1A1; 2 Consumer and Clinical Radiation Protection Bureau, Health Canada, Ottawa, Ontario, Canada K1A 1C1; 3 The University of Texas MD Anderson Cancer Center, Houston, TX 77030, USA

**Keywords:** EGFR, triazene, DNA, quinazoline

## Abstract

Recently, with the purpose of enhancing the potency of epidermal growth factor receptor (EGFR)-based therapies, we designed a novel strategy termed ‘Cascade-release targeting’ that seeks to develop molecules capable of degrading to multiple tyrosine kinase (TK) inhibitors and highly reactive electrophiles, in a stepwise fashion. Here we report on the first prototype of this model, RB24, a masked methyltriazene, that in addition to being an inhibitor on its own was designed to degrade to RB14, ZR08, RB10+a DNA alkylating methyldiazonium species. The cascade degradation of RB24 requires the generation of two reactive electrophiles: (a) an iminium ion and (b) a methyldiazonium ion. Thus, we surmise that these species could alkylate the active site of EGFR, thereby irreversibly blocking its action and that DNA damage could be induced by the methyldiazonium. Using the EGFR-overexpressing human epidermoid carcinoma of the vulva cell line, A431, we demonstrate herein that (a) RB24 and its derived species (e.g. RB14, ZR08) irreversibly inhibit EGFR autophosphorylation, (b) RB24 induced significant levels of DNA strand breaks, (c) sustained inhibition of EGFR by RB24 was associated with blockade of MAPK activation and c-fos gene expression, (d) RB24 induced irreversible cell growth inhibition with a 100-fold greater potency than Temodal™, a clinical methyltriazene. The pronounced growth inhibitory potency of RB24 was attributed to its ability to simultaneously damage DNA and irreversibly block EGFR TK activity.

Overexpression of the epidermal growth factor receptor (EGFR) family and its cognate ligand has been correlated with aggressiveness and poor prognosis in various tumours such as breast, ovarian and prostate cancer (Modjtahedi and Dean, 1994; Xie *et al*, 1995; Turner *et al*, 1996; Meden and Kuhn, 1997; Chen *et al*, 2000). The implication of these receptors in cancer progression has garnered significant attention and agents capable of blocking disordered growth signalling mediated by these proteins are now in a significant number of clinical trials against many cancers. One such agent, Iressa, a trademark of the AstraZeneca group of companies, exhibits a broad spectrum of antitumour activity against many human solid tumour xenografts of various origins including breast, lung, colorectal and head and neck (Ciardiello *et al*, 2001a; Moulder *et al*, 2001; Heimberger *et al*, 2002; Magné *et al*, 2002; Ranson *et al*, 2002). However, despite the significant activity of this compound in preclinical models, in Phase II clinical trial, it only induced a response rate of approximately 10% in a cohort of patients with non-small-cell lung cancer (NSCLC) (Magné *et al*, 2002; Dancey and Freidlin, 2003). Several possible explanations have been put forth to explain the failure to demonstrate a benefit: inadequate dosing, reduced drug delivery to tumour, lack of sustained potency and failure to select patients on the basis of having tumours in which EGFR presents a growth advantage (Dancey and Freidlin, 2003).

The lack of sustainability of the antitumour action of reversible inhibitors has stimulated the design of new irreversible inhibitors of EGFR tyrosine kinase (TK). One such compound, PD183805, bearing a 6-acrylamido group designed to alkylate cysteine 773 in the active site of the ATP-binding pocket irreversibly blocks EGFR TK and is now in Phase I development in patients with head and neck, breast and non-small cell lung carcinoma (Ciardiello and Tortora, 2001b). Other approaches to enhance the potency of EGFR inhibitor-based therapy include combinations of inhibitors with several cytotoxic agents including taxol, cytoxan and adriamycin (Ciardiello *et al*, 1999, 2001a; Magné *et al*, 2002; Dancey and Freidlin, 2003). Within the same line of idea, with the purpose of developing more potent and targeted therapies, we developed a novel strategy termed ‘Combi-targeting’ that seeks to synthesise single molecules that are capable of both blocking EGFR TK and inducing cytotoxicity by damaging DNA (Brahimi *et al*, 2002; Matheson *et al*, 2001, 2003a, 2004a; Qiu *et al*, 2003, 2004; Rachid *et al*, 2003). Two such compounds, SMA41 (Matheson *et al*, 2003a, 2004b) and BJ2000 (Brahimi *et al*, 2002), the first models designed to demonstrate the feasibility of this principle, showed significant DNA-damaging ability and irreversible block of EGFR autophosphorylation in A431 cells. More importantly, these agents induced a more sustained antiproliferative activity when compared with a reversible inhibitor of EGFR (Matheson *et al*, 2001, 2004b; Brahimi *et al*, 2002). In addition, these molecules, also termed ‘Combi-molecules,’ selectively induced antiproliferative activity against EGFR transfectants in isogenic models (Brahimi *et al*, 2002; Matheson *et al*, 2003a).

In order to further augment the potency of the Combi-targeting approach, we recently designed RB24 (an acetoxymethyltriazene) to generate three inhibitors of EGFR and at the final stage of degradation a methyldiazonium species capable of damaging DNA (Banerjee *et al*, 2003). This masked cluster of molecules was expected to produce more sustained antitumour effects with the prospect of inducing activities similar or superior to that of classical combinations involving a reversible EGFR inhibitor+a cytotoxic drug. Here we study the mechanism of action of RB24, the first prototype of this strategy termed ‘cascade release’, and demonstrate the sustainability of its antitumour activity in A431 cells, which overexpress EGFR and its cognate ligand, TGF-*α* (Lanzi *et al*, 1997). The ability of this cell line to aggressively proliferate by a TGF-*α*-mediated autocrine induction has made it an ideal model for studying the mechanism of action of EGFR TK inhibitors. In addition, these cells express *O*6-alkylguanine transferase (*O*6-AGT), a DNA repair enzyme that by repairing the *O*6-alkylguanine DNA adduct confers significant resistance to AGT+ cells (Lee *et al*, 1991; Mitchel and Dolan, 1993; Pegg *et al*, 1995; Cai *et al*, 2000; Yingna *et al*, 2000; Matheson *et al*, 2003b).

## MATERIALS AND METHODS

### Drug treatment

RB24, RB14, ZR08 and RB10 were reported elsewhere (Banerjee *et al*, 2003). Temodal™ was provided by Shering-Plough Inc. (Kenilworth, NJ, USA). In all assays, the drug was dissolved in DMSO and subsequently diluted in RPMI-1640 containing 10% fetal bovine serum (FBS) (Wisent Inc., St-Bruno, Canada) immediately before the treatment of cell cultures. In all assays, the concentration of DMSO never exceeded 0.2% (v/v).

### Cell culture

The cell line used in this study, the human epidermoid carcinoma of the vulva, A431, was obtained from the American Type Culture Collection (Manassas, VA, USA). The A431 cell line was maintained in RPMI-1640 supplemented with 10% FBS and antibiotics as described previously (Matheson *et al*, 2001). All cells were maintained in an atmosphere of 5% CO_2_.

### *In vitro* cytokinetic growth inhibition assay

To study the irreversible effects of our compounds on cell proliferation, 200 cells well^−1^ were plated in 96-well plates with serum-containing media. Cells were exposed to each drug+serum for either 2, 8, 12, 24 or 48 h. After each time point, the drug was removed and cells were washed 2 × with PBS and allowed to recover with fresh serum-containing media for a total of 96 h. Cell growth was measured using the sulphorhodamine B (SRB) assay. Briefly, following drug treatment and recovery, cells were fixed using 50 *μ*l of cold trichloroacetic acid (50%) for 60 min at 4°C, washed five times with tap water and stained for 30 min at room temperature with SRB (0.4%) dissolved in acetic acid (0.5%). The plates were rinsed five times with 1% acetic acid and allowed to air dry. The resulting coloured residue was dissolved in 200 *μ*l of Tris base (10 mM), and optical density was read for each well at 492 nm using a Bio-Rad microplate reader (model 2550). Each point represents the average of at least two independent experiments run in triplicate.

### Autophosphorylation assay

A431 cells (1 × 10^6^) were preincubated in a six-well plate with 10% serum at 37°C for 48 h and starved overnight for 24 h, after which they were exposed to a dose range of each drug for 2 h and subsequently treated with 50 ng ml^−1^ EGF for 15 min at 37°C. Cells were washed with PBS and resuspended in cold lysis buffer (50 mM Tris-HCl pH 7.5, 150 mM NaCl, 1% Nonidet P-40, 1 mM EDTA, 5 mM NaF, 1 mM Na_3_VO_4_, protease inhibitor tablet (Roche Biochemicals, Laval, Canada)). The lysates were kept on ice for 30 min and collected by centrifugation at 10 000 rpm for 20 min at 4°C. The concentrations of protein were determined using the Bio-Rad protein assay kit (Bio-Rad Laboratories, Hercules, CA, USA). Equal amounts of protein were added to a 10% SDS–polyacrylamide gel electrophoresis (SDS–PAGE) and transferred to a polyvinylidene difluoride membrane (Millipore, Bedford, MA, USA). Nonspecific binding on the membrane was minimised with a blocking buffer containing nonfat dry milk (5%) in PBST. Thereafter, the membranes were incubated with primary antibodies (either antiphosphotyrosine antibody (Upstate Biotechnology, Lake Placid, NY, USA) for the detection of phosphotyrosine, or anti-EGFR (Neomarkers, Fremont, CA, USA)) for determination of corresponding receptor levels. Blots were incubated with HRP-goat anti-mouse antibody (Bio-Rad Laboratories) and the bands visualised with an enhanced chemiluminescence system (Amersham Pharmacia Biotech, Buckinghamshire, UK). Band intensities were measured using the SynGene GeneTools software package.

To study the effects of RB24 on the activation of extraceullular signal-regulated kinases 1,2 (Erk1,2), protein lysates were obtained as described above and Western blot was performed as previously reported (Tari and Lopez-Berestein, 2000). The membrane was incubated with antiphosphorylated Erk1,2 antibodies or antibodies specific for Erk1,2 (Cell Signaling, Beverly, MA, USA).

### Reverse EGFR autophosphorylation

This assay was performed as previously described (Fry *et al*, 1998). A431 cells were grown to confluence in six-well plates and then incubated in serum-free medium for 24 h. Duplicate sets of cells were then treated with 30 *μ*M of each compound for 90 min. One set of cells was then stimulated with EGF (50 ng ml^−1^) for 15 min and extracts were made as described under the Western blotting procedure above. The other set of cells was washed free of the compound with warm serum-free media and incubated for 2 h. Thereafter, the cells were washed, incubated for another 2 h, washed again and then incubated for a further 4 h. This set of cells was then stimulated with EGF and extracts were prepared as for the first set.

### RT*–*PCR for c-fos expression

A431 cells were grown to confluence in six-well plates and then incubated in serum-free medium for 24 h. Cells were exposed to the indicated concentrations of the drug prior to stimulation with EGF (50 ng ml^−1^) for 30 min. Total RNA was isolated using the High Pure RNA Isolation Kit of Roche Molecular Bochemicals (Germany), following the manufacturer's instructions. Quantitative analysis of c-fos mRNA and G3PDH mRNA (2 *μ*g of RNA for each sample) was preformed by Titan One Tube RT–PCR Kit (Roche Molecular Bochemicals), following the manufacturer's instructions and using the following primers: 5′ATGATGTTCTCGGGCTTC3′ (sense), 5′CTCCTGCCAATGCT CTGC3′ (antisense) for c-fos and 5′CCATGGAGAAGGCTGGGG3′ (sense), 5′CAAA GTTGTCATGGATGACC3′ (antisense) for G3PDH.

### Alkaline comet assay for quantitation of DNA damage

The alkaline comet assay was performed as previously described (Matheson *et al*, 2001). The cells were exposed to drugs (RB24, RB14 or RB10) for 30 min, harvested with trypsin–EDTA, subsequently collected by centrifugation and resuspended in PBS. Cell suspensions were diluted to approximately 10^6^ cells and mixed with agarose (1%) in PBS at 37°C in a 1 : 10 dilution. The gels were cast on Gelbond strips (Mandel Scientific, Guelph, Canada) using gel-casting chambers, as previously described (McNamee *et al*, 2000), and then immediately placed into a lysis buffer (2.5 M NaCl, 0.1 M tetrasodium EDTA, 10 mM Tris-base, 1% (w/v) *N*-lauryl sarcosine, 10% (v/v) DMSO and 1% (v/v) Triton X-100, pH 10.0). After being kept on ice for 30 min, the gels were gently rinsed with distilled water and immersed in a second lysis buffer (2.5 M NaCl, 0.1 M tetrasodium EDTA, 10 mM Tris-base) containing 1 mg ml^−1^ proteinase K for 60 min at 37°C. Thereafter, they were rinsed with distilled water, incubated in alkaline electrophoresis buffer for 30 min at 37°C and electrophoresed at 300 mA for 20 min. The gels were subsequently rinsed with distilled water and placed in 1 M ammonium acetate for 30 min. Thereafter, they were soaked in 100% ethanol for 2 h, dried overnight and stained with SYBR Gold (1 : 10 000 dilution of stock supplied from Molecular Probes, Eugene, OR, USA) for 20 min. Comets were visualised at × 330 magnification and DNA damage was quantitated using the Tail Moment parameter (i.e. the distance between the barycentre of the head and the tail of the comet multiplied by the percentage of DNA within the tail of the comet). A minimum of 50 cell comets were analysed for each sample, using ALKOMET version 3.1 image analysis software.

## RESULTS

### Inhibition of EGFR autophosphorylation

**Figure 1:**
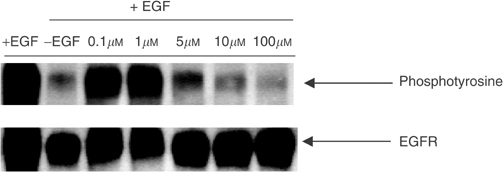
Selective inhibition of EGFR autophosphorylation in intact cells by RB24. Serum-starved A431 cells were preincubated for 2 h with the indicated concentrations of RB24 prior to stimulation with EGF for 15 min. An equal amount of cell lysates was analysed by Western blotting using antiphosphotyrosine antibodies. Membranes were stripped of antiphosphotyrosine and reprobed with anti-EGFR antibodies as a loading control. Band intensities were measured using the SynGene GeneTools software package.

**Figure 2:**
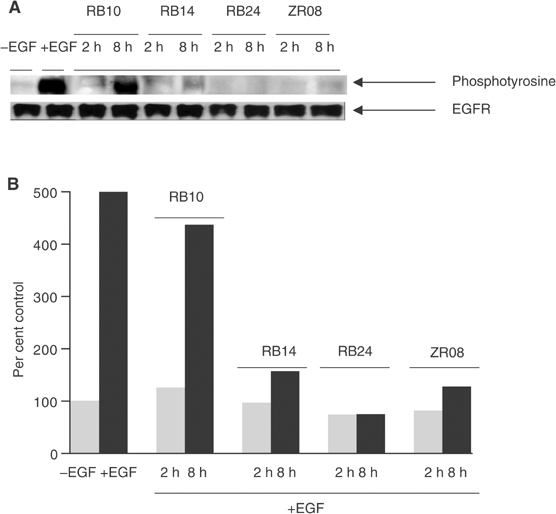
Reverse EGFR autophosphorylation in the presence of RB24, RB14, ZR08 and RB10 in A431 cells. (**A**) Duplicate sets of cells were treated with 30 *μ*M of designated compound to be tested as a reversible EGFR inhibitor for 90 min. One set of cells was then stimulated with EGF for 15 min, and extracts were made as described under the Western blotting procedure. The other set of cells was washed free of the compound with serum-free media, incubated for 2 h, and further washed twice and incubated for 4 h. This set of cells was then stimulated with EGF, and extracts were made similar to the first set. (**B**) Comparison between the inhibition of autophosphorylation activity induced by RB24, RB14, ZR08 and RB10. The film was scanned and band intensities were quantified using Syngene GeneTools software. Values are percentage of control of phosphotyrosine/EGFR.

Western blot analysis demonstrated that RB24 blocked EGF-induced EGFR autophosphorylation in A431 cells in a dose-dependent manner with an IC_50_≈2 *μ*M without affecting the levels of EGFR (Figure 1

). RB24 is a degradable molecule capable of generating an extremely reactive iminium ion intermediate that may alkylate the receptor and irreversibly inhibit EGFR activity. To test this hypothesis, we used the reversibility assay previously described (Fry *et al*, 1998; Smaill *et al*, 2000) according to which the cells were treated with the drug for 90 min and the culture medium repeatedly removed and replaced three times after treatment, after which EGFR autophosphorylation was measured. As expected, RB24 at 30 *μ*M completely suppressed EGF-dependent EGFR autophosphorylation in A431 cells immediately after drug exposure (Figure 2A

). However, at 8 h post-treatment following repeated washouts in drug-free medium, EGFR autophosphorylation activity was completely inhibited in cells treated with RB24, indicating that the latter is capable of inducing irreversible inhibition of EGFR autophosphorylation. Similarly, irreversible inhibition was observed in cells exposed to the daughter molecules, RB14 and ZR08, however with a 20% recovery of the total activity (Figure 2B). In contrast, the naked inhibitor, RB10, was shown to have completely reversible inhibition of EGFR autophosphorylation (Figure 2A).

### Inhibition of EGFR-mediated signalling

**Figure 3:**
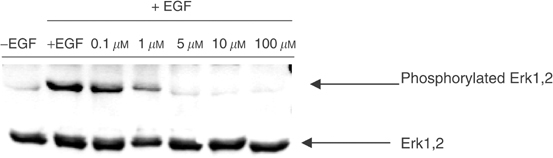
Effect of RB24 on Erk1,2 activation in A431 cells. Serum-starved cells were preincubated for 2 h with the indicated concentrations of RB24 prior to stimulation with EGF for 15 min. Protein lysates were obtained and Western blot was performed as described by Tari and Lopez-Berestein (2000).

**Figure 4:**
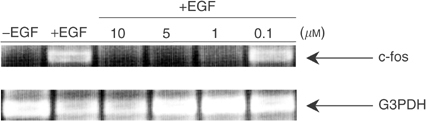
Effect of RB24 on c-fos gene expression in A431 cells. Serum-starved cells were preincubated for 2 h with the indicated concentrations of RB24 prior to stimulation with EGF for 30 min. Quantitative analysis of c-fos and G3PDH was preformed by RT–PCR as described in Materials and methods.

Antiproliferative activity induced by RB24 requires the translation of inhibition of EGFR autophosphorylation into inhibition of downstream signalling. To determine whether blockade of EGFR autophosphorylation translates into inhibition of downstream signalling, we analysed the effect of the parent compound, RB24, on EGF-induced phosphorylation of Erk1,2 and c-fos expression in A431 cells. The results showed that RB24 induced complete inhibition of Erk1,2 phosphorylation at concentrations as low as 5 *μ*M without affecting the levels of Erk1,2 (Figure 3

). Similarly, RT–PCR analysis showed that RB24 induced nearly 100% inhibition of EGF-mediated c-fos gene expression at low concentrations (1 *μ*M) (Figure 4

), indicating that inhibition of EGFR phosphorylation by RB24 is accompanied by a significant blockade of EGFR-dependent downstream signalling.

### Quantitation of DNA damage

**Figure 5:**
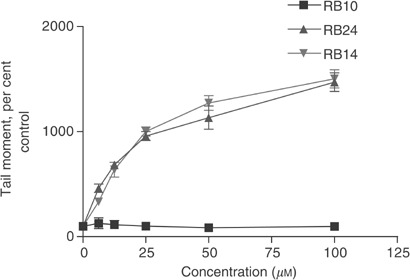
Quantitation of DNA damage using the alkaline comet assay. Tail moment was used as a parameter for the detection of DNA damage in A431 cells exposed to RB24, RB14 and RB10 for 30 min. Each point represents at least two independent experiments.

Using the alkaline comet assay, we demonstrated that like TEM (Matheson *et al*, 2001), RB24 and RB14 induced a dose-dependent DNA damage in A431 cells after a 30 min drug exposure (Figure 5

). Interestingly, RB24 and RB14 induced identical levels of DNA damage, which is in agreement with the fact that the former is a prodrug of the latter. As expected, the reversible inhibitor, RB10, did not demonstrate any DNA-damaging activity.

### Irreversible growth inhibitory activity

**Figure 6:**
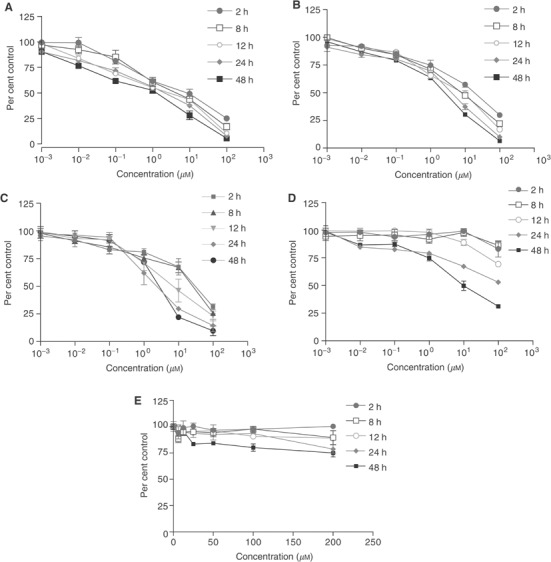
Irreversible growth inhibition for RB24, RB14, ZR08, RB10 and TEM in A431 cells. Cells were exposed to (**A**) RB24, (**B**) RB14, (**C**) ZR08, (**D**) RB10 or (**E**) TEM for 2, 8, 12, 24 or 48 h following recovery for a total of 96 h. Cell growth was measured using SRB assay. Each point represents at least two independent experiments run in triplicate.

SRB assays demonstrated that RB24 and RB14 retained significant antiproliferative activity after a short 2 h exposure and a 4-day recovery (Figure 6A and B

). In contrast, the free inhibitor, RB10, lost at least 95% of its activity under the same conditions (Figure 6D). The monoalkyltriazene, ZR08, showed partially reversible activity with an 85% retention of activity following 2 h drug exposure (Figure 6C). In contrast to the antiproliferative effects of RB24, increasing exposure time was associated with a significant increase in potency for all other molecules of the degradation cascade (RB14, ZR08, RB10). Despite being a potent alkylating agent, the clinical triazene, Temodal™, demonstrated no significant activity at any of the exposure times in the A431 cells (Figure 6E). In summary, the strength of the retention of potency was in the following order: RB24>RB14>ZR08≫RB10.

## DISCUSSION

### Mechanisms of EGFR TK inhibition

**Scheme 1:**
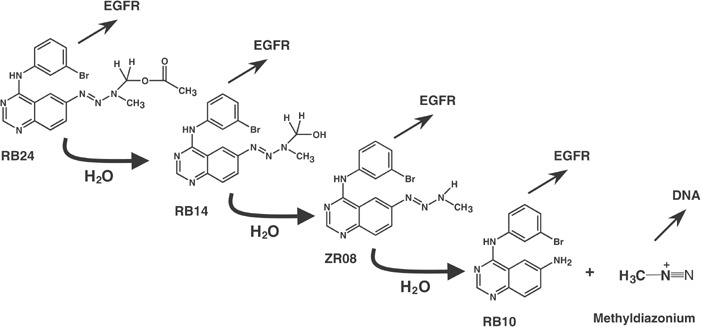

**Scheme 2:**
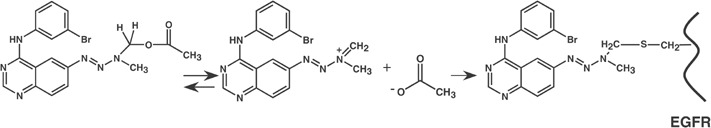

The design of our combi-molecule, RB24, was based on the premise that acetoxymethyltriazenes are known to be hydrolysed to a hydroxymethyltriazene intermediate that rapidly degrades into the corresponding monoalkyltriazene (Hemens *et al*, 1984; Cameron *et al*, 1985; Hemens and Vaughan, 1986). The latter further heterolyses to an aromatic amine+a DNA-damaging species. In a recent publication (Banerjee *et al*, 2003), we demonstrated that indeed RB24 was capable of generating the ultimate amino compound RB10, in a symmetrically inversed relationship. As depicted in Scheme 1

, decomposition through the intermediates, RB14 and ZR08, is the sole mechanism by which RB24 can be converted to RB10. Thus, the significant EGFR inhibitory potency of the putative intermediates RB14, ZR08 and RB10 indicates that RB24 would maintain its TK inhibitory potency throughout the multistep degradation cascade. More importantly, RB24 and its two derivatives (RB14, ZR08) induced irreversible inhibition of EGFR TK when tested alone, a protracted inhibition that may be rationalised in the light of recent data on the mechanism of irreversible acrylamide-based inhibitors. It is now known that 4-anilinoquinazolines bearing a 6-acrylamido group react with the cysteine 773 of EGFR, thereby irreversibly blocking the receptor (Fry *et al*, 1998; Smaill *et al*, 1999, 2000). Based on the mechanism of decomposition of RB24 (Banerjee *et al*, 2003), we did not expect a direct reaction between the cysteine thiol group and the acetoxy moiety. Perhaps, if the iminium ion is formed in the active site of EGFR, it may react with the cysteine as depicted in Scheme 2

. Addition of thiol group to the iminium ions of acetoxymethyltriazene has already been reported by Iley *et al* (1991) who developed this type of triazenes as lyase inhibitors. Moreover, the ultimate triazene metabolite of RB24, ZR08, may also directly alkylate the cysteine residue. Quinazolinotriazenes of the same class, BJ2000 and SMA41, have already been demonstrated to induce irreversible inhibition (Brahimi *et al*, 2002; Matheson *et al*, 2003b, 2004b).

Although it was not the focus of this study to identify the site of alkylation of the receptor, the irreversible nature of TK inhibition by RB24 indicates that it may have inflicted covalent damage at a site of EGFR that is critical for its TK activity. In corroboration, RB10 that does not possess a reactive triazene tail induced reversible EGFR TK inhibitory activity.

### DNA damage

The iminium ion is a stable cation that may not be reactive enough to account for the strong DNA-damaging potential of RB24. Despite the significant body of results that confirm its formation during the process of hydrolysis of acetoxymethyltriazenes (Hemens *et al*, 1984; Hemens and Vaughan, 1986; Iley, 1987; Vaughan and Manning, 1988; Merrin and Hooper, 1992), its implication in the DNA-damaging properties of the latter class of compounds is yet to be demonstrated. In contrast, it is now common knowledge that the methyldiazonium is capable of inducing significantly high levels of DNA alkylation particularly at N7 and O6 positions of guanine, thereby inducing DNA damage and lethal mutations in tumour cells (Bodell *et al*, 1985; Tisdale, 1987; Baer *et al*, 1993). The promutagenic *O*6-alkylguanine adduct is considered to be the primary cytotoxic lesion induced by triazenes. Cells, like A431, that express high levels of AGT, a DNA repair enzyme that repairs the *O*6-alkylguanine adduct, are known to be resistant to alkyltriazenes. In corroboration, the A431 cells used in the study were markedly insensitive to Temodal™, a cyclic triazene known to release the methyldiazonium species upon hydrolysis (Cameron *et al*, 1985; Gibson *et al*, 1986; Baig and Stevens, 1987).

### Irreversible growth inhibition

The ability of RB24 to degrade into several species with significant EGFR inhibitory activities and to damage DNA may perhaps be responsible for its sustained potency in A431 cells. More importantly, while its antiproliferative effect was maintained even 4 days after a 2 h drug exposure, under the same conditions, ZR08 induced a partially reversible growth inhibitory activity. We have already reported similar results in A431 cells for analogous quinazolinotriazenes (e.g. SMA41 and BJ2000). Thus, the marked irreversibility of the action of RB24 may be partially imputed to additive antiproliferative contribution of species generated during the degradation steps that precede the formation of ZR08. This antiproliferative contribution may result from their ability to block EGFR-mediated growth signalling, since as previously mentioned the transient iminium ion species generated during these degradation steps may not damage DNA. The partially reversible growth inhibitory potency of ZR08 (third step of the cascade) may perhaps be due to the dependence of its antiproliferative activity on the ultimately released reversible inhibitor RB10, the concentrations of which might have been drastically depleted by multiple washouts. This indicates that the ultimate antiproliferative effect of ZR08 is mediated by a combination of DNA damage inflicted by the methyldiazonium species and EGFR TK inhibitory activity induced by RB10.

A synergistic interaction between the strong EGFR TK inhibitory potency and the marked DNA-damaging potential of RB24 may also partly account for its remarkable potency against the A431 cells. Indeed, blockade of EGFR in these cells was accompanied by potent inactivation of Erk1,2 kinase and inhibition of c-fos gene expression. We now know, based on previous experiments, that blockade of EGFR-mediated signalling may not affect the levels or function of the AGT enzymes. However, Yacoub *et al* (2003) recently demonstrated that EGF upregulates the DNA repair genes XRCC1 and ERCC1 in prostate cell lines through Erk1,2 signalling. Thus, blockade of EGFR-mediated signalling may downregulate the DNA repair enzymes that are involved in the repair of DNA strand breaks, thereby exacerbating the cytotoxic effect of the latter lesions.

In conclusion, we have demonstrated that a molecule engineered to possess complex EGFR inhibitory properties and capable of further releasing DNA-damaging species could induce significantly more sustained antiproliferative activity than a reversible inhibitor of the same class (e.g. RB10). Moreover, its multiple properties have conferred antiproliferative activity in a cell line in which a classical methylating agent of the same class does not show any detectable activity. Thus this model may well represent a new strategy to ameliorate the chemotherapy of EGF-dependent refractory tumours. Further studies are ongoing to demonstrate the potency of this novel drug *in vivo*.
